# The influence of type-I and type-II triplet multiple quantum well structure on white organic light-emitting diodes

**DOI:** 10.1186/1556-276X-8-529

**Published:** 2013-12-17

**Authors:** Bo Zhao, Zisheng Su, Wenlian Li, Bei Chu, Fangming Jin, Xingwu Yan, Tianyou Zhang, Feng Zhang, Di Fan, Yuan Gao, Junbo Wang, Huachun Pi, Jianzhuo Zhu

**Affiliations:** 1 State Key Laboratory of Luminescence and Applications, Changchun Institute of Optics, Fine Mechanics, and Physics, Chinese Academy of Sciences, Changchun 130033, People’s Republic of China; 2 University of Chinese Academy of Sciences, Beijing 100039, People’s Republic of China; 3 Key Laboratory of Optical System Advanced Manufacturing Technology, Changchun Institute of Optics, Fine Mechanics and Physics, Chinese Academy of Sciences, Changchun 130033, People’s Republic of China; 4 Sinosteel Scie-tech Development Co. Ltd, 8-Haidian Street, Beijing 100080, People’s Republic of China; 5 College of Science, Yanshan University, Qinhuangdao 066004, People’s Republic of China

**Keywords:** White organic light-emitting diodes, Organic quantum well structure, Type-I and type-II, Potential barrier layer

## Abstract

We demonstrate high-efficient white organic light-emitting diodes (WOLEDs) based on triplet multiple quantum well (MQW) structure and focus on the influence on WOLEDs through employing different potential barrier materials to form type-I and type-II MQWs, respectively. It is found that type-I MQW structure WOLEDs based on 1,3,5-tris(*N*-phenyl-benzimidazol-2-yl)benzene as potential barrier layer (PBL) offers high electroluminescent (EL) performance. That is to say, maximum current efficiency and power efficiency are achieved at about 1,000 cd/m^2^ with 16.4 cd/A and 8.3 lm/W, which increase by 53.3% and 50.9% over traditional three-layer structure WOLEDs, respectively, and a maximum luminance of 17,700 cd/m^2^ is earned simultaneously. The achievement of high EL performance would be attributed to uniform distribution and better confinement of carriers within the emitting layer (EML). However, when 4,7-diphenyl-1,10-phenanthroline or 2,9-dimethyl-4,7-diphenyl-1,10-phenanthroline is used as PBL to form type-II MQW structure, poor EL performance is obtained. We attribute that to improper energy level alignment between the interface of EML/PBL, which leads to incomplete confinement and low recombination efficiency of carriers, a more detailed mechanism was argued.

## Background

Compared to inorganic light-emitting diodes (LEDs), which have developed for several decades and are still being researched [1-3], organic light-emitting diodes (OLEDs) now have also attracted intensive attention due to their bright future on practical application [4,5]. In recent years, white organic light-emitting diodes (WOLEDs) have become a research highlight; because of their potential applications in solid-state lighting, panel display technology etc., various WOLEDs constructions have been demonstrated [6-9]. Among the structures, multiple quantum well (MQW) device is one of the significant white emission devices because charge carriers and excitons could be confined in a narrow emissive zone to prevent the emitter from interacting with the adjacent emitter, which is highly similar to the working mechanism of the inorganic MQW constitution of LED. MQW is generally divided into type-I and type-II configurations in OLEDs. Type-I MQW structure is defined as the narrow bandgap molecule located within the wide bandgap molecule; thus, injected carriers are confined between the lowest unoccupied molecular orbital (LUMO) and the highest occupied molecular orbital (HOMO) energy levels of the narrow bandgap molecule. While the LUMO/HOMO energy levels of both two materials in type-II MQW structure are staggered, carriers are confined in different molecules.

WOLEDs with the MQW structure have been reported, thanks to the confinement of carriers and excitons within potential wells, but their emissive efficiency is generally lower than that of the traditional three-layer structure. For example, Xie et al. and Yang et al. had respectively fabricated an MQW structure white device, but both efficiencies of the fabricated structures were low [10,11]. The reason for the low efficiency of those MQW structure WOLEDs are attributed to the use of fluorescent material only and incomplete confinement of charge carriers and excitons within the emitting layer (EML) due to adoption of undeserved potential barrier layer (PBL) materials. In order to improve the emissive efficiency of the MQW structure, triplet phosphor must be used and PBL also needs to be skillfully used. Our group had designed triplet MQW structure WOLEDs in which 1,3,5-tris(*N*-phenyl-benzimidazol-2-yl)benzene (TPBi) was used as PBL, and blue fluorescent dye and orange phosphor doped EML were used as two potential well layers (PWLs), respectively [12]. As a result of the application of better PBL and triplet emitter component PWLs, a peak luminance of 19,000 cd/m^2^ and a current efficiency of 14.5 cd/A were achieved. During the process of researching the triplet MQW structure, it is found that the PBL material played a key role in improving the performance of WOLEDs, and the distribution of carriers with different PBL materials was also different, but it seemed that such issues have rarely been taken into account.

In light of the mentioned argument, we continued the investigation on triplet MQW structure in this manuscript to further develop an active design of MQW structure WOLEDs. Here, TPBi was used as the PBL, and 4,4′-*N*,*N*′-dicarbazole-biphenyl (CBP) was adopted as the host, 4,4′-bis(9-ethyl-3-carbazovinylene)-1,1′-biphenyl (BCzVBi) was used as blue fluorescent dopant, and fac-tris(2-phenylpyridine) iridium(III) (Ir(ppy)_3_) and tris(1-phenylisoquinoline)iridium(III) (Ir(piq)_3_) were used as green and red phosphor dopants, respectively. It was found that the WOLEDs with TPBi as the PBL formed type-I MQW structure and showed the best electroluminescent (EL) performance, i.e., maximum luminance, peak current efficiency, and power efficiency are 17,700 cd/m^2^, 16.4 cd/A, and 8.3 lm/W, which increased by 53.3% and 50.9% for current efficiency and power efficiency compared to those in a traditional three-layer structure, respectively. The improved EL performance was attributed to uniform distribution and rigorous confinement of carriers and excitons. We also constructed WOLEDs with type-II MQW structure, in which the PBL of TPBi in the above-mentioned WOLEDs was changed to 4,7-diphenyl-1, 10-phenanthroline (Bphen) or 2,9-dimethyl-4,7-diphenyl-1, 10-phenanthroline (BCP), respectively, but keeping other condition to be identical. Low EL performances were obtained, which resulted from poor confinement of carriers and excitons within the EML of the type-II MQW structure; a more detailed mechanism was also discussed.

## Methods

Patterned indium tin oxide (ITO)-coated glass substrates with a sheet resistance of 10 Ω/sq were routinely cleaned and treated with ultraviolet ozone for 15 min before loading into a high vacuum chamber (approximately 3 × 10^−4^ Pa). The organic materials for fabrication were procured commercially without further purification. Thermal deposition rates for organic materials, metal oxide, and Al were 0.2, 0.05, and 1 nm/s, respectively. Al cathode was finally deposited with a shadow mask that defined an active device area of 3 × 3 mm^2^. The WOLEDs were with the following structure: ITO/MoO_3_ (5 nm)/CBP (20 nm)/CBP: 10% BCzVBi (5 nm)/PBL (2 nm)/CBP: 5% Ir(ppy)_3_ (4 nm)/PBL (2 nm)/CBP: 4% Ir(piq)_3_ (4 nm)/PBL (2 nm)/Bphen (45 nm)/LiF (1 nm)/Al (100 nm). Here, PBL denotes TPBi, Bphen, and BCP for devices A, B, and C, respectively; MoO_3_, CBP, and Bphen function as hole injection layer, hole transport layer, and electron transport layer, respectively; doped EMLs of blue, green, and red act as PWLs simultaneously in MQW structure WOLEDs. The device without PBL is referred to as reference device with the traditional three-layer structure. EL spectra were measured with an OPT-2000 spectrophotometer (Photoelectric Instrument Factory of Beijing Normal University, Beijing, China). The current–voltage-luminance characteristics were measured with a Keithley (Cleveland, OH, USA) 2400 power supply combined with ST-900 M spot photometer (Photoelectric Instrument Factory of Beijing Normal University, Beijing, China) and were recorded simultaneously with measurements. All measurements were carried out at room temperature and under ambient conditions without any protective coatings.

## Results and discussion

**Figure 1:**
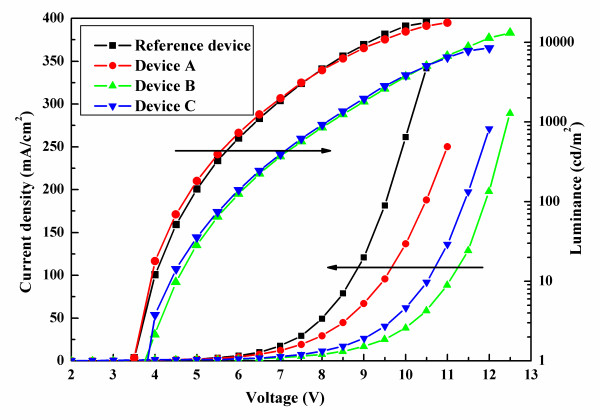
Current density-voltage-luminance characteristics of all WOLEDs.

**Figure 2:**
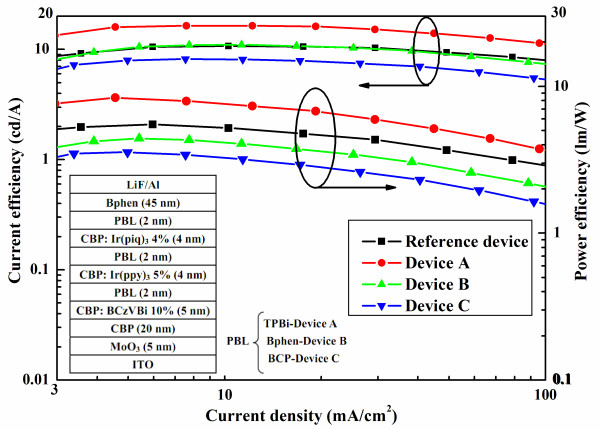
**Current efficiency-current density-power efficiency characteristics of all WOLEDs.** Inset: the device structures.

**Figure 3:**
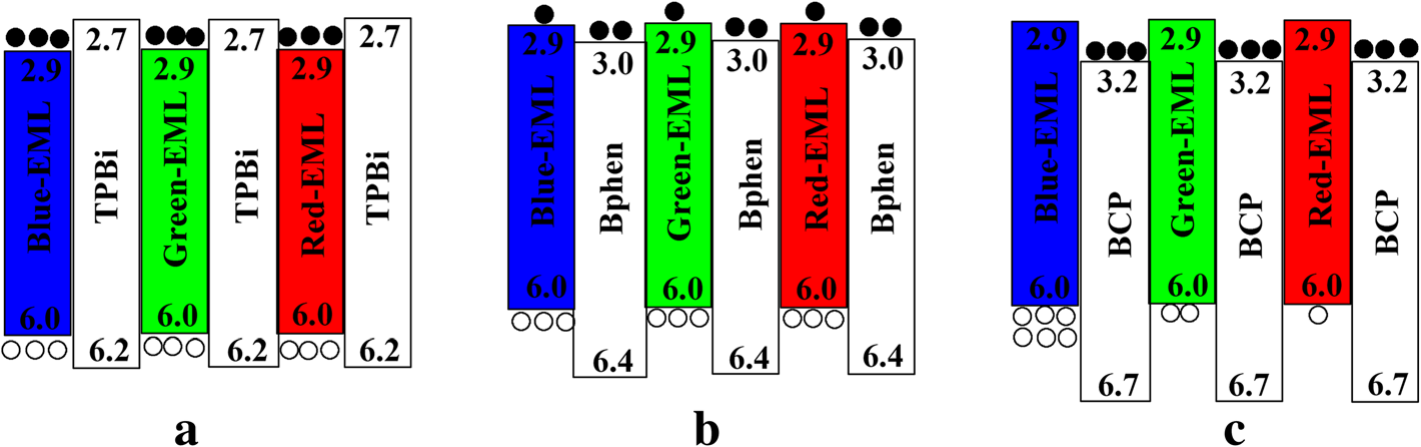
**The schematic energy level diagram of WOLEDs with the portion of EMLs. (a)** device A. **(b)** device B. **(c)** device C. Black circle and white circle express electron and hole, respectively. The numbers indicate the LUMO and HOMO energies relative to vacuum (in eV). Here, LUMO and HOMO are cited from [18-20].

**Figure 4:**
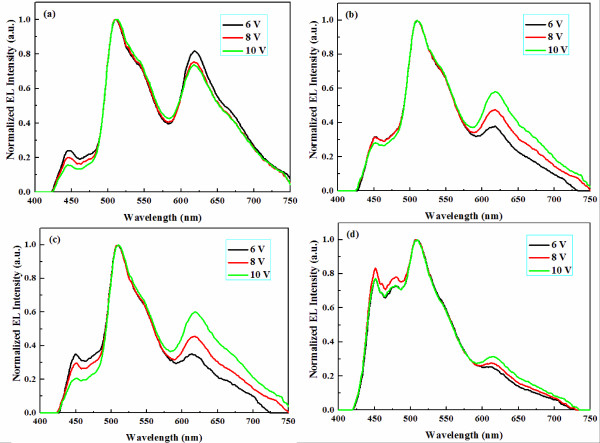
**The EL spectra of all WOLEDs under various voltages. (a)** Reference device, **(b)** device A, **(c)** device B, and **(d)** device C.

Figure 1 exhibits the characteristics of current density-voltage-luminance. The reference device has a maximum current density at the same voltage due to the absence of PBL. Figure 2 shows the current efficiency-current density-power efficiency characteristics of all WOLEDs, and the inset depicts the device structures. Device A exhibits a maximum current efficiency of 16.4 cd/A and power efficiency of 8.3 lm/W at about 1,000 cd/m^2^, which are higher than those of the reference device by 53.3% and 50.9%, respectively. It is noted that the EL performance of the reference device with CBP as the host of blue, green, and red emissions is almost identical to international reported results [13-15]. That is to say, the reference device in this paper is an optimum performance, which could be used to contrast. Furthermore, we also see that the Commission International de I'Eclairage (CIE) coordinates here are better than those of the reference device due to a lower *x* value (see Table 1). Thus, we consider that the type-I MQW structure is in favor of achieving a higher EL performance than the traditional three-layer structure. This can be understood as follows: for device A with type-I MQW structure, injected electrons and holes located at potential wells as EMLs and the barriers at the interface of EML/TPBi are 0.2 eV either at the LUMO or HOMO energy level, which can be seen in Figure 3a. Under external electrical field, electrons and holes are injected from the cathode and anode, respectively, then the carriers would overcome the 0.2-eV barriers to enter into EML, and the uniform distribution and balanced recombination of carriers in all EMLs could take place. Another improved factor is the confinement of triplet excitons within EMLs because the triplet energy of TPBi is 2.74 eV [16], which is higher than that of CBP, Ir(ppy)_3_, and Ir(piq)_3_ which are 2.56 [17], 2.41, and 2.0 eV, respectively. Therefore, PBL of TPBi also has the function of exciton blocking, which can confine excitons efficiently within each EML and prevent them from migrating to adjacent EML. In contrast, because of the absence of PBL and the host is entirely CBP in the reference device, electrons and holes can be transported without any barriers. Singlet excitons produced in blue EML would partly be transferred to green EML to result in a week emission of blue light. Also, the triplet excitons in green EML could also be transferred into red EML so that strong red emission is observed, as shown in Figure 4a. Such exciton transfers above must lead to the poor EL performance of the reference device.

**Table 1:** Summary of EL performance of all WOLEDs in this study

	***V***_**on**_^**a**^**(V)**	**CE**_**max**_^**b**^**(cd/A)**	**PE**_**max**_^**c**^**(lm/W)**	**CE**^**d**^**at 1,000 cd/m**^**2**^**(cd/A)**	**PE**^**e**^**at 1,000 cd/m**^**2**^**(lm/W)**	**CIE at 10 V ( *x *, *y *)**
Reference device	3.52	10.7	5.5	10.6	5.2	(0.38, 0.45)
Device A	3.56	16.4	8.3	16.2	8.1	(0.32, 0.45)
Device B	3.76	11.0	4.4	10.9	4.2	(0.32, 0.45)
Device C	3.82	8.1	3.5	8.0	3.1	(0.24, 0.35)

^a^Turn-on voltage; ^b^maximum current efficiency; ^c^maximum power efficiency; ^d^current efficiency at 1,000 cd/m^2^; ^e^power efficiency at 1,000 cd/m^2^.

Another two MQW structure WOLEDs have low efficiencies compared to device A, even lower than that of the reference device. Devices A, B, and C offer a peak luminance of 17 700, 13,200, and 8,489 cd/m^2^, respectively. The difference between luminances indicates the different recombination efficiencies because luminance is generally decided by the recombination degree between electrons and holes [21]. Table 1 summarizes the EL performances of all devices. Such a large difference between their EL performances could be understood from different alignments between LUMO/HOMO energy levels of EML/PBL due to the use of different PBL materials. First, let us see the schematic energy level diagrams of WOLEDs with the portion of EMLs that are shown in Figure 3. Device A with TPBi as PBL belongs to the foregoing type-I MQW structure, and LUMO/HOMO energy levels (bandgap) of each EML located within LUMO/HOMO energy levels of TPBi and two carriers are confined in the EML, while devices B and C belong to the type-II MQW structure with Bphen and BCP as PBL, respectively. The LUMO/HOMO energy levels of PBL and EML are staggered, and only a single carrier is confined in the EML. For device A, there is a 0.2-eV barrier at the interface of either [LUMO]_EML_/[LUMO]_TPBi_ or [HOMO]_EML_/[HOMO]_TPBi_, and such an energy level alignment makes electrons and holes distribute uniformly in the EMLs that act as potential wells under electrical excitation. All the electrons and holes could be confined in EMLs due to the presence of a suitable energy level of TPBi, which would increase a recombination possibility between the two carriers and produce more excitons in EML [22]. For device B, the potential well of holes is the EML with a 0.4-eV barrier at the [HOMO]_EML_/[HOMO]_Bphen_ interface; injected holes could easily be confined within the HOMO energy level of EML. However, there is only a 0.1-eV barrier that lies in the [LUMO]_EML_/[LUMO]_Bphen_ interface, so electrons seem to cross the barrier easily and distribute all throughout the LUMO energy level of EML and Bphen, so less electrons could be present in the LUMO energy level of EML, as shown in Figure 3b, which leads to a low recombination efficiency. For device C, the situation is similar to device B, as indicated in Figure 3c. However, there is a 0.3-eV barrier at the [LUMO]_EML_/[LUMO]_BCP_ interface, and electrons are confined in the LUMO energy level of BCP. Meanwhile, the larger barrier of 0.7 eV at the interface of [HOMO]_EML_/[HOMO]_BCP_ results in holes confined in the HOMO energy level of EML. Since electrons and holes are confined in different organic layers, which increase the probability of excitons disassociation and decrease the recombination efficiency of carriers [23], device C presents inferior EL performances.

Therefore, the different level alignments both for [LUMO]_EML_/[LUMO]_PBL_ and [HOMO]_EML_/[HOMO]_PBL_ for devices A, B, and C lead to the different distributions and recombination efficiencies of carriers. That is also proven by their different EL spectra as shown in Figure 4. From the emission spectra, we note that device A with type-I MQW structure offers a larger blue emission than the reference device which makes better CIE coordinates (see Table 1). For devices B and C with type-II MQW structure, there is a low possibility of carrier recombination due to the fact that only a single carrier could be confined in the EML, while another carrier is confine in PBL, which results in poor EL performances. It is a fact that strong blue emission and week red emission present in device C resulted from the accumulation of holes at the interface of [HOMO]_blue-EML_/[HOMO]_BCP_ and that there are less holes in potential wells of green EML and red EML, especially in potential wells of red EML.

## Conclusions

In conclusion, WOLEDs with type-I MQW structure offer higher EL performances in contrast with the reference device with traditional three-layer structure. WOLEDs with TPBi as PBL exhibits a peak current efficiency and a power efficiency of 16.4 cd/A and 8.3 lm/W at about 1,000 cd/m^2^, which increase by 53.3% and 50.9% over the reference device, respectively; meanwhile, a maximum luminance of 17,700 cd/m^2^ is achieved, which keeps a similar luminance with the reference device. The achievement of high EL performance with type-I MQW structure WOLEDs would be attributed to the uniform distribution and rigorous confinement of carriers and excitons within EMLs. However, when Bphen or BCP acts as PBL instead of TPBi, low EL performances (especially for BCP) are obtained, which are attributed to poor level alignment at the interface of EML/PBL for type-II MQW structure; thus, incomplete confinement and low recombination efficiency of carriers occur. In terms of the results, we find that type-I MQW is a promising structure design for improving white EL performance by choosing the suitable PBL. It is also expected that feasible energy level alignment between EML and PBL, suitable depth of potential well, and high triplet energy of PBL would be beneficial to demonstrate high-performance WOLEDs with MQW structure.

## Abbreviations

EL: Electroluminescent; EML: Emitting layer; HOMO: Highest occupied molecular orbital; ITO: Indium tin oxide; LUMO: Lowest unoccupied molecular orbital; MQW: Multiple quantum well; PBL: Potential barrier layer; PWL: Potential well layer; WOLEDs: White organic light-emitting diodes.

## Competing interests

The authors declare that they have no competing interests.

## Authors’ contributions

BZ wrote the manuscript and carried out the experiments and data analysis. ZSS, WLL, and BC guided the experiment’s progress and manuscript writing and participated in mechanism discussions. FZ, DF, JBW, HCP, and JZZ took part in mechanism discussions. FMJ, XWY, TYZ, and YG helped measure and collect the experimental data. All authors read and approved the final manuscript.
